# Verification of Atellica 1500 and comparison with Iris urine analyser and urine culture

**DOI:** 10.11613/BM.2022.010701

**Published:** 2021-12-15

**Authors:** Ana Nikler, Helena Čičak, Danijela Bejuk, Vanja Radišić Biljak, Ana-Maria Šimundić

**Affiliations:** 1 Department of Medical Laboratory Diagnostics, University Hospital “Sveti Duh”, Zagreb, Croatia; 2 Department of Clinical Microbiology and Hospital Infections, University Hospital “Sveti Duh”, Zagreb, Croatia; 3 Faculty of Pharmacy and Biochemistry, University of Zagreb, Zagreb, Croatia

**Keywords:** Atellica 1500 analyser, Iris analyser, urine, verification

## Abstract

**Introduction:**

The aims of study were to assess: 1) performance specifications of Atellica 1500, 2) comparability of Atellica 1500 and Iris, 3) the accuracy of both analysers in their ability to detect bacteria.

**Materials and methods:**

Carryover, linearity, precision, reproducibility, and limit of blank (LoB) verification were evaluated for erythrocyte and leukocyte counts. ICSH 2014 protocol was used for estimation of carryover, CLSI EP15-A3 for precision, and CLSI EP17 for LoB verification. Comparison for quantitative parameters was evaluated by Bland-Altman plot and Passing-Bablok regression. Qualitative parameters were evaluated by Weighted kappa analysis. Sixty-five urine samples were randomly selected and sent for urine culture which was used as reference method to determine the accuracy of bacteria detection by analysers.

**Results:**

Analytical specifications of Atellica 1500 were successfully verified. Total of 393 samples were used for qualitative comparison, while 269 for sediment urinalysis. Bland-Altman analysis showed statistically significant proportional bias for erythrocytes and leukocytes. Passing-Bablok analysis for leukocytes pointed to significant constant and minor proportional difference, while it was not performed for erythrocytes due to significant data deviation from linearity. Kappa analysis resulted in the strongest agreements for pH, ketones, glucose concentrations and leukocytes, while the poorest agreement for bacteria. The sensitivity and specificity of bacteria detection were: 91 (59-100)% and 76 (66-87)% for Atellica 1500 and 46 (17-77)% and 96 (87-100)% for Iris.

**Conclusion:**

There are large differences between Atellica 1500 and Iris analysers, due to which they are not comparable and can not be used interchangeably. While there was no difference in specificity of bacteria detection, Iris analyser had greater sensitivity.

## Introduction

The urine analysis is one of the most commonly performed tests in medical laboratories. It is an important tool in diagnostic pathway for various urinary tract disorders (*1*). Various guidelines for the assessment of chronic kidney disease, urinary tract infections and tumor formations emphasize the importance of urinalysis as the first step in diagnostic pathway and further evaluation of patient condition (*2*-*4*).

Automation of urine analysis has been a major step forward in urine analysis, by standardizing the analysis and substantially reducing the analysis turnaround time (*5*, *6*). Over the past years, automated urine analysis has become an inevitable part of routine medical laboratory practice. Automated urine analysers employ different method principles. Atellica 1500 (Siemens healthineers, Erlangen, Germany) is one of the most recent automated urine analysers, which uses a built-in centrifuge and a camera to capture sediment images that are evaluated by a sophisticated software for classification of urine particles. The performance of Iris (Beckman Coulter, Brea, USA) urine analyser differs from Atellica 1500. Iris method is based on laminar flow digital imaging technology without previous sample centrifugation. Currently there are no studies about verification of new Atellica 1500 specifications and its comparison to Iris analyser. According to their methods differences it is questionable whether the urine results of these two analysers are even comparable. A further challenge is their accuracy and agreement with the urine culture in capability of bacteria detection.

Therefore, the aims of our study were to assess: 1) performance specifications of Atellica 1500 urine analyser: carryover, linearity, precision, reproducibility and verification of limit of blank (LoB) for erythrocyte and leukocyte counts, 2) comparability of Atellica 1500 and Iris urine analyser, and 3) the accuracy of both analysers in their ability to detect bacteria.

## Material and methods

The study was performed at Department of Medical Laboratory Diagnostics of University hospital “Sveti Duh” (Zagreb, Croatia) during July 2018.

### Methods comparison

Leftover urine samples consecutively collected from emergency and routine hospital departments during daily routine were used to estimate comparison of two urine analysers: Atellica 1500 (Siemens healthineers, Erlangen, Germany) and Iris (Beckman Coulter, Brea, USA). Samples were analysed within 1 hour after their collection. Atellica 1500 analyser is consisted of Clinitek Novus (urine strip analysis) and Atellica UAS 800 (automated urine sediment analyser).

Dipstick parameters included in this study were: pH, ketones, glucose, leukocytes, nitrites, bilirubin, blood, specific gravity, proteins and urobilinogen, while yeasts, mucus, hyaline casts, epithelial cells, bacteria, erythrocyte and leukocyte count were included in urine sediment analysis.

Comparison for quantitative parameters (erythrocyte and leukocyte counts) was evaluated by Bland-Altman plot and Passing-Bablok regression. Cusum test was used to detect the deviation of data from linearity. In cases when deviation from linearity was detected, Passing-Bablok analysis was not done. Qualitative parameters were evaluated by Weighted kappa analysis.

Weighted kappa test was used to determine levels of agreement for urine test strips analysis and following urine sediment parameters: yeast, mucus, hyaline casts, epithelial cells and bacteria. The agreement was expressed as Cohen kappa value (κ). Kappa value ≥ 0.60 was considered acceptable (*7*). Default manufacturer’s reporting categories were used. For the purpose of the agreement assessment, some categories were merged to gain at least 10 samples *per* category.

Urine test strips parameters which were analysed as negative and positive and their respective cut-offs are as follows: ketones (2 mmol/L), nitrites (16.2 µmol/L), glucose (14 mmol/L), bilirubin (8.5 µmol), urobilinogen (33 µmol/L) and proteins (0.5 g/L). Urine pH was categorized into three categories, as follows: 5.0-5.5, 6.0-6.5, 7.0-7.5 and 8.0-9.0 units, while specific gravity categories were: 1.000-1.009, 1.010-1.019 and > 1.020. Blood and leukocytes were divided in three categories: ≤ 33, ≤ 163, ≤ 326 Erc/µL and ≤ 25, ≤ 75, ≤ 500 Lkc/µL. Urine sediment parameters (modified due to merging, as described above) are shown in Table 1.

**Table 1:** Categories of urine sediment parameters used to compare Atellica UAS 800 (Siemens healthineers, Erlangen, Germany) and Iris (Beckman Coulter, Brea, USA) urine analysers

**Urine sediment parameter (p/µL)**	**1^st^ category**	**2^nd^ category**	**3^rd^ category**
yeast	Iris 0-11.0 Atellica 0-4.49	Iris ≥ 11.1 Atellica ≥ 4.50	N/A
mucus	Iris 0-27.5 Atellica 0-396	Iris ≥ 27.6 Atellica ≥ 397	N/A
hyaline casts	Iris 0-0.7 Atellica 0-2.97	Iris ≥ 0.8 Atellica ≥ 2.98	N/A
epithelial cells	Iris 0-27.5 Atellica 0-7.52	Iris 27.6-55.0 Atellica 7.53-37.49	Iris ≥ 55.0 Atellica ≥ 37.50
bacteria	Iris 0-6.8 Atellica 0-195.03	Iris 6.9-13.8 Atellica 195.04-495	Iris ≥ 13.9 Atellica ≥ 496
Concentration ranges of categories were defined by manufacturer. p/µL – particles *per* microliter of urine. N/A – not available.			

Data were analysed using MedCalc 12.6.2.0 (Ostend, Belgium) statistical software and Microsoft Office Excel 2010 (Microsoft, Washington, USA).

### Accuracy of the ability to detect bacteria in urine

Sixty-five urine samples that were analysed on both urine analysers were randomly selected and sent for urine culture analysis within 20 minutes after urine sediment analysis. Those urine samples were analysed in Microbiology Department of our hospital. The urine culture was used as reference method to determine the accuracy of bacteria detection by Atellica UAS 800 and Iris analysers.

Each urine sample was cultured for organism quantification, identification of bacteria and susceptibility testing. Urine culture quantification was performed according to the standard practice using 10 microliter calibrated loops. After inoculating the loopful of urine to the blood agar plate on a standard way, plates were incubated overnight at 35 °C. In the urine culture, the number of colony-forming units (CFUs) *per* mL is an estimate of the number of bacteria in the sample. If uropathogens (Gram negative rods, *Staphylococcus saprophyticus, Enterococcus*) were isolated full identification and antimicrobial testing were done. If skin urinary microbiota was isolated (diphteroides, coagulase negative *Staphylococcus spp*) in pure culture and in significant number (> 10^5^ CFU/mL) identification and antimicrobial testing was also performed.

To determine the accuracy of bacteria detection by Atellica UAS 800 and Iris urine analysers, a bacterial count of > 10^5^ CFU/mL was considered as positive bacterial urine culture result, regardless of whether it was Gram negative or positive bacteria. The sensitivity and specificity of both analysers to detect bacteria in urine samples were calculated as showed in Table 2. The sensitivity was calculated as “true positives” (TP) / (TP + ”false negatives” (FN)), while specificity as “true negatives” (TN) / (TN + “false positives” (FP)).

**Table 2:** Contingence table of determining sensitivity and specificity of Atellica UAS 800 (Siemens healthineers, Erlangen, Germany) and Iris (Beckman Coulter, Brea, USA) urine analysers for bacteria detection compared to urine culture as reference method

			**urine culture**		
			**positive**	**negative**	**∑**
urine analyser		positive	TP	FP	TP + FP
		negative	FN	TN	FN + TN
	**∑**		TP + FN	FP + TN	Total
TP - true positive. FP - false positive. FN - false negative. TN - true negative.					

Atellica UAS 800 and Iris analysers have different reporting categories for urine sediment results. Therefore, the results were provided in absolute numbers and in categories declared by both manufacturers.

### Carryover

The carryover was estimated for leukocyte and erythrocyte counts in urine sediment analysis in accordance with ICSH 2014 protocol (*8*, *9*). The carryover testing was performed using a sample with a high count (sample A) and another one with a low count (sample B) of leukocytes and erythrocytes. The sample A was analysed three times (A1, A2, A3), followed by the sample B that was also analysed three times (B1, B2, B3). The carryover (%) was calculated using the equation (B1 - B3) / (A3 - B3), wherein the carryover < 0.5% was considered as acceptable.

### Linearity, precision, reproducibility and verification of LoB

Linearity, precision, reproducibility and verification of LoB were determined for quantitative urine sediment parameters, *i.e.* erythrocyte and leukocyte counts.

The linearity was assessed on two urine samples with high leukocyte and erythrocyte counts. Samples were diluted with distilled water in 1:0, 1:2, 1:4, 1:8 and 1:16 ratios. The observed values were plotted against the expected values wherein R^2^ > 0.99 was considered as linear and acceptable.

Precision was determined according to CLSI EP15-A3 using quality control materials (qUAntify Plus Control, BioRad, LOT 80561 and 80562, expiration date 30^th^ November 2019) (*10*). These two control materials were analysed five times *per* day during five consecutive days. According to manufacturer’s declarations, acceptable criteria for erythrocytes was 14% and 11% for leukocytes. Two patient urine samples with high and low number of erythrocytes and leukocytes were also analysed 20 times to estimate the reproducibility on patient samples.

Limit of blank was verified according to CLSI EP17 guideline (*11*). Ten blank samples (distilled water) *per* day were analysed for 3 days. Limit of blank values for erythrocyte and leukocyte counts declared by manufacturer were 0 Erc/µL and 0 Lkc/µL.

### Determination of different sample volume effect

To determine whether a different volume of urine sample affects the results of erythrocyte and leukocyte counts, we decided to analyse 10 mL as maximum volume sample as many times as Atellica 1500 can analyse it without alarm for insufficient sample. Each time analyser has taken small amount of sample for analysis. Four randomly selected urine samples were used, and the recovery percentage was calculated for each sample.

## Results

### Atellica 1500 and Iris comparison

Total number of urine samples analysed on Iris analyser with urine test strips was 393. Urine samples without any abnormalities in urine test strips were not subjected to urine sediment analysis. Thus, sediment urinalysis was determined for 269/393 urine samples.

The results of kappa statistical analysis of agreement between Atellica 1500 (Clinitek Novus) and Iris for test strip and urine sediment analysis are presented in Table 3. The strongest agreements were observed for the following test strip parameters: pH, ketones, glucose concentrations and leukocytes count. The poorest agreement was observed for bacteria in urine sediment.

**Table 3:** The agreement of Atellica 1500 (Siemens healthineers, Erlangen, Germany) and Iris (Beckman Coulter, Brea, USA) automated urine analysers determined by Cohen kappa value (κ) for urine test strips and sediment analysis

	**Parameter**	**Kappa value (κ)**	**Level of agreement**	**Acceptance**
**Urine test strips**	pH	0.88	strong	yes
	Ketones	0.84	strong	yes
	Glucose	0.82	strong	yes
	Leukocytes	0.80	strong	yes
	Nitrites	0.77	moderate	yes
	Bilirubin	0.72	moderate	yes
	Blood	0.61	moderate	yes
	Specific gravity	0.59	weak	no
	Proteins	0.51	weak	no
	Urobilinogen	0.08	none	no
**Urine sediment**	Yeast	0.75	moderate	yes
	Mucus	0.61	moderate	yes
	Hyaline casts	0.45	weak	no
	Epithelial cells	0.45	weak	no
	Bacteria	0.20	low	no

**Figure 1:**
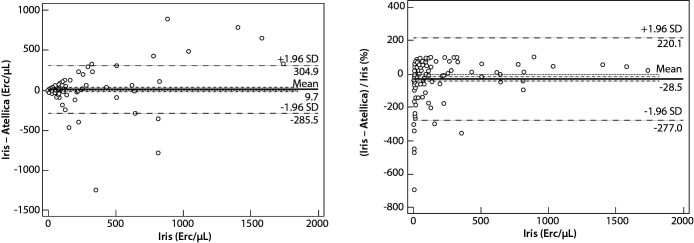
Bland-Altman plots of absolute and relative differences showing the differences in erythrocytes counts (Erc/µL) between Atellica UAS 800 (Siemens healthineers, Erlangen, Germany) and Iris (Beckman Coulter, Brea, USA) urine analysers. Solid line – mean difference. Dotted lines - 95% confidence interval of mean difference. Dashed lines – ±1.96 standard deviation (SD).

**Figure 2:**
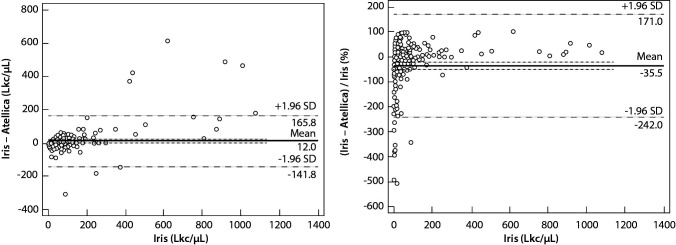
Bland-Altman plots of absolute and relative differences showing the differences in leukocytes counts (Lkc/µL) between Atellica UAS 800 (Siemens healthineers, Erlangen, Germany) and Iris (Beckman Coulter, Brea, USA) urine analysers. Solid line – mean difference. Dotted lines - 95% confidence interval of mean difference. Dashed lines – ±1.96 standard deviation (SD).

**Figure 3:**
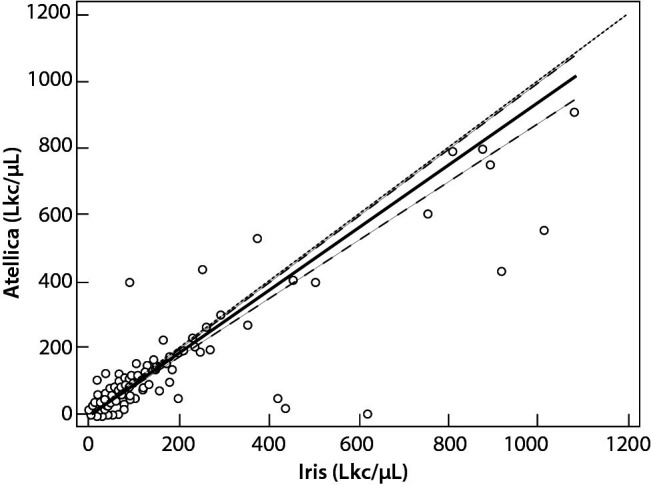
Passing and Bablok regression plots comparing leukocytes counts (Lkc/µL) on Atellica UAS 800 (Siemens healthineers, Erlangen, Germany) and Iris (Beckman Coulter, Brea, USA). Solid line - regression line. Dashed line – 95% confidence interval of the regression line. Dotted line - identity line (y=x). y = 1.38 (0.99 to 2.13) + 0.93 (0.87 to 0.99) x.

Bland-Altman agreement analysis showed statistically significant proportional bias for erythrocytes and leukocytes in urine sediment (Figures 1, 2). Due to significant data deviation from linearity (P < 0.010), Passing-Bablok regression analysis for erythrocyte count was not performed. Passing-Bablok regression analysis for leukocytes pointed to the significant constant and minor proportional difference in leukocyte count in urinary sediment, between Atellica UAS 800 and Iris automated urine analyser (Figure 3).

### Accuracy of bacteria detection compared to urine culture

The sensitivity and specificity of bacteria detection were: 91 (59-100)% and 76 (66-87)% for Atellica UAS 800 and 46 (17-77)% and 96 (87-100)% for Iris (Table 4). There was a statistically significant difference in analysers specificity, but not in the sensitivity of their bacteria detection. The results of bacteria detection comparison by Iris and Atellica 1500 urine analysers compared to urine culture results are showed in Supplementary Table 1.

**Table 4:** Contingence table of determining sensitivity and specificity for bacteria detection of Atellica UAS 800 (Siemens healthineers, Erlangen, Germany) and Iris (Beckman Coulter, Brea, USA) urine analysers

			**urine culture**		
			**positive**	**negative**	**∑**
**Atellica** **UAS 800**		positive	10	13	23
		negative	1	41	42
		**∑**	11	54	65
			**urine culture**		
			**positive**	**negative**	**∑**
**Iris**		positive	5	2	7
		negative	6	52	58
		**∑**	11	54	65
Urine culture was used as reference method. TP - true positive. FP - false positive. FN - false negative. TN - true negative.					

#### Other Atellica 1500 specifications

No carryover effect was observed for erythrocytes and leukocytes counts.

**Figure 4:**
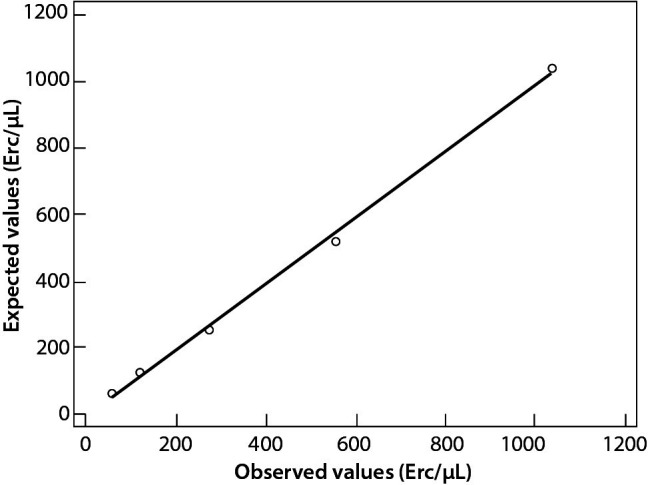
Linearity plot of observed and expected erythrocyte counts (Erc/µL) on Atellica UAS 800 (Siemens healthineers, Erlangen, Germany) analyser. Coefficient of determination (R^2^) was 0.9978.

**Figure 5:**
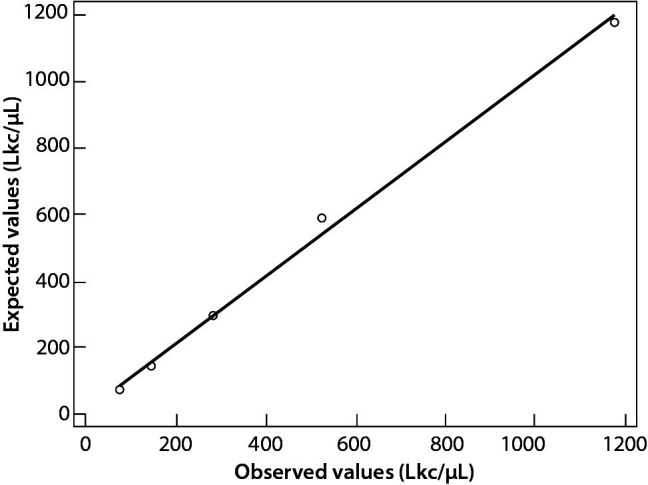
Linearity plot of observed and expected leukocyte counts (Lkc/µL) on Atellica UAS 800 (Siemens healthineers, Erlangen, Germany) analyser. Coefficient of determination (R^2^) was 0.9967.

Erythrocyte counts used to evaluate linearity were in the range of 62-1034 Erc/µL along with 80-1174 Lkc/µL leukocytes. Average bias for erythrocytes count was 1.87%, and 0.004% for leukocytes. The coefficients of determination were within acceptance criteria of R^2^ > 0.99: 0.9978 for erythrocytes and 0.9967 for leukocytes (Figures 4, 5).

Method precision at 187.16 Erc/µL was 7.87% for erythrocytes, and 8.91% at 108.19 Lkc/µL for leukocytes. Coefficients of variations (CVs) for both urine parameters were within acceptance criteria. Negative control materials contained erythrocytes and leukocytes below detection limits (< 4 Erc/µL and < 3 Lkc/µL) in each measurement and therefore no statistical analysis can be performed. For within-run reproducibility CVs were 7.94% for 194.44 Erc/µL and 9.25% for 110.42 Lkc/µL.

Limit of blank for erythrocytes and leukocytes declared by manufacturer was successfully verified. All measurements of erythrocyte and leukocyte counts were below detection limits (< 4 Erc/µL and < 3 Lkc/µL).

### Sample volume influence on urine analysis results

Four urine samples used to examine whether the sample volume affects the results of urine analysis were successfully analysed seven times. For each sample, the eighth analysis was not able to proceed because the analyser notified that there was not enough sample volume. Erythrocytes and leukocytes counts and recovery percentages of each sample were presented in Table 5.

**Table 5:** Recovery percentages and following erythrocytes and leukocytes counts of four urine samples used to examine the effect of sample volume on urine analysis results

**Erythrocytes**		**Leukocytes**	
**Count** **(Erc/µL)**	**Recovery** **(%)**	**Count** **(Lkc/µL)**	**Recovery (%)**
12.32	- 5.95	60.06	- 1.83
53.68	- 14.46	95.70	- 15.20
199.76	- 5.43	252.12	4.49
228.80	10.17	900.00	1.17

## Discussion

Our study has demonstrated large differences between Iris and Atellica 1500 automated urine analysers that use different methodologies. Two analysers differ in their comparability of urine test strips parameters, especially for specific gravity, proteins and urobilinogen while differences in urine sediment parameters were even more pronounced.

The disagreement of urine test strips analysis among different manufacturers has been already demonstrated, as well as discrepancy of urinalysis by automated urine analysers (*12*-*14*). Some previous studies based on comparison of various automated urine analysers have already highlighted major differences in their specifications and performances (*14*-*17*). Therefore, a certain level of disagreement of urine analysers developed by different manufacturers, based on diverse particle detection methods, was expected for this study.

Barbir *et al.* provided comparison of Atellica UAS 800 (Siemens healthineers, Erlangen, Germany) and Cobas u701 (Roche, Rotkreuz, Switzerland) analysers on a much smaller number of urine samples, in comparison to our study (*18*). They observed constant and proportional bias for erythrocyte and leukocyte counts, which is in accordance with results of our study. We have proved statistically significant constant bias for leukocytes and proportional bias for both erythrocytes and leukocytes. They also compared Atellica 1500 with microscopic urine sediment analysis using supravital staining which is an advantage over our study. Comparing to microscopic analysis they revealed proportional bias for erythrocytes and both constant and proportional bias for leukocytes counts. But our study encompassed a more detailed evaluation of Atellica 1500 specifications in addition to the comparison of Atellica 1500 with another urine analyser in their erythrocytes and leukocytes detection. We have successfully verified the following performance specifications of Atellica 1500 urine analyser: carryover, linearity, precision, reproducibility and LoB verification for erythrocyte and leukocyte counts. Additionally, the main advantage of our study is examined accuracy of Atellica UAS 800 and Iris urine analysers in their ability to detect bacteria.

Urinalysis is the first step in patient diagnostic pathway, where clinicians based on the following urine test strips and sediment results decide about further patient diagnostic examination and treatment. Along with the clinical symptoms of infections and presence of leukocytes in urine samples, bacteriuria is one of the main signs of urinary tract infections (UTIs) (*3*). Patients with present bacteriuria detected by routine urinalysis need to be sent for further urine culture. Foudraine *et al.* assessed great utility of iQ200 (Beckman Coulter, Brea, USA) parameters in combination with clinical symptoms and urine culture to predict or rule out UTIs (*19*). The number of urine culture analysis depends on bacteria results obtained by urine analysers. Therefore, it is of utmost importance to precisely and accurate detect bacteria. The accuracy of bacteria detection of Atellica UAS 800 and iQ200 analysers comparing with urine culture results showed intriguing results. Statistically significant difference in specificity of bacteria detection by these two urine analysers was observed. Iris analyser more specifically detects bacteria comparing to Atellica. But there was no statistically significant difference in their sensitivity of bacteria detection. Accurate bacteria detection is important to minimize the number of false positives and limit inappropriate antibiotic use.

During the performance of this study, we have noticed an inconsistency among urine analysers that may represent a potential source of aggravated interpretation of urine analysis results. The great discrepancy of reference ranges suggested by manufacturers was obtained. For example, negative bacteria results were < 6.8 p/µL for Iris, and < 195.03 p/µL for Atellica. It is important that clinicians are familiar with this problem so that they can properly interpret results of urinalysis and provide adequate medical care of patients.

The absence of manual microscopic urine test results is a possible limitation of this study.

In conclusion, Atellica 1500 urine analyser meets manufacturer’s quality criteria and has satisfactory analytical performance. There are large differences between Atellica 1500 and Iris analysers, due to which these two instruments are not comparable and can not be used interchangeably. Additionally, while there was no difference in specificity of bacteria detection between these two urine analysers, Iris analyser had greater sensitivity in bacteria detection.
